# Computational Approaches for Microalgal Biofuel Optimization: A Review

**DOI:** 10.1155/2014/649453

**Published:** 2014-09-21

**Authors:** Joseph Koussa, Amphun Chaiboonchoe, Kourosh Salehi-Ashtiani

**Affiliations:** Division of Science and Math and Center for Genomics and Systems Biology (CGSB), New York University Abu Dhabi, P.O. Box 129188, Abu Dhabi, UAE

## Abstract

The increased demand and consumption of fossil fuels have raised interest in finding renewable energy sources throughout the globe. Much focus has been placed on optimizing microorganisms and primarily microalgae, to efficiently produce compounds that can substitute for fossil fuels. However, the path to achieving economic feasibility is likely to require strain optimization through using available tools and technologies in the fields of systems and synthetic biology. Such approaches invoke a deep understanding of the metabolic networks of the organisms and their genomic and proteomic profiles. The advent of next generation sequencing and other high throughput methods has led to a major increase in availability of biological data. Integration of such disparate data can help define the emergent metabolic system properties, which is of crucial importance in addressing biofuel production optimization. Herein, we review major computational tools and approaches developed and used in order to potentially identify target genes, pathways, and reactions of particular interest to biofuel production in algae. As the use of these tools and approaches has not been fully implemented in algal biofuel research, the aim of this review is to highlight the potential utility of these resources toward their future implementation in algal research.

## 1. Introduction

Biofuel production from microalgae has been receiving attention as an alternative energy source due to its high biomass productivity and minimal land resource requirement. However, there is still a need to improve algal productivity in order to make algal-based bioproducts economically viable. Metabolic network reconstructions of algae can offer insight into genetic modification strategies that can be used to improve microalgal strains. A large number of computational tools have been developed, allowing a range of analyses and predictions, based on genetic and thermodynamic constraints embedded in in the network, to identify bioengineering strategies that can result in enhanced biofuel production of the engineered algal strain. Although a fair number of algal genomes have been fully sequenced, only a few metabolic network models have been reconstructed for these species, hampering algal bioengineering progress [1].

The utilities of metabolic network models span over several types of applications. On one hand, these models help contextualizing high throughput experimental data, for example, integrating gene expression data with metabolic pathways under different growth conditions [2]. Metabolic models can also unveil targets for metabolic engineering approaches, which can lead to increased production of target metabolites [3] or preferentially increase respiration rates [4]. On the other hand, with the availability of large and diverse biological data sets, metabolic network models can provide a framework to integrate such omics data and allow the formulation and testing of downstream hypotheses. Last, cross-species metabolic comparison represents one more utility of such reconstructions through which identification of differentially activated metabolic pathways can be achieved among other comparative analyses [5]. Herein we review the reconstruction of metabolic network models and major computational tools and pipelines that hold the potential to contribute to the optimization of algal strains for biofuel production. We describe a number of tools that remain mostly unused by the algal research community. This is reflected from the observation that only 7 algal-based PGDBs (Pathway/Genome Database) are available in Pathway Tools [6], while approximately 3,500 PGDBs are available for nonalgal species (please see below for more information). The use of some of the herein discussed tools, already applied to the multitude nonalgal organisms, ranging from human to* E. coli*, provides strategies for algal biofuels optimization with major enhancement potential.

## 2. Metabolic Network Model Reconstruction

Metabolic network reconstruction from genomic and large-scale experimental data can help understand and predict metabolic processes and pathways. A number of tools and databases have been developed specifically to facilitate metabolic network reconstruction. In addition, new analysis tools and approaches are being developed along with the expansion of relevant databases and resources.  Table 1 presents some of the existing databases and tools for metabolic network reconstruction.

Metabolic network reconstruction requires information on gene-protein-reaction associations to reconstruct evidence-based, species-specific networks. Protein database resources and tools help to link information between enzymes, EC numbers, genes, proteins, pathways, and substrates. These include BRENDA [7], ExPASy [8], and UniProt (Universal Protein Resource) [9]. BRENDA (BRaunschweig ENzyme DAtabase) enzyme portal is the enzyme information system, which integrates information from seven databases to provide functional biochemical and molecular data. To explore and visualize metabolic networks as maps of metabolic pathways, a number of freely available pathway databases exist. For example, BioCyc, MetaCyc [10], KEGG (Kyoto Encyclopedia of Genes and Genomes) [11], Reactome [12], and BiGG [13] can be named. In turn, common metabolic reconstruction tools include COBRA (more specifically its rBioNet component) [14–16], Model SEED [17], and Pathway Tools [6].

**Figure 1:**
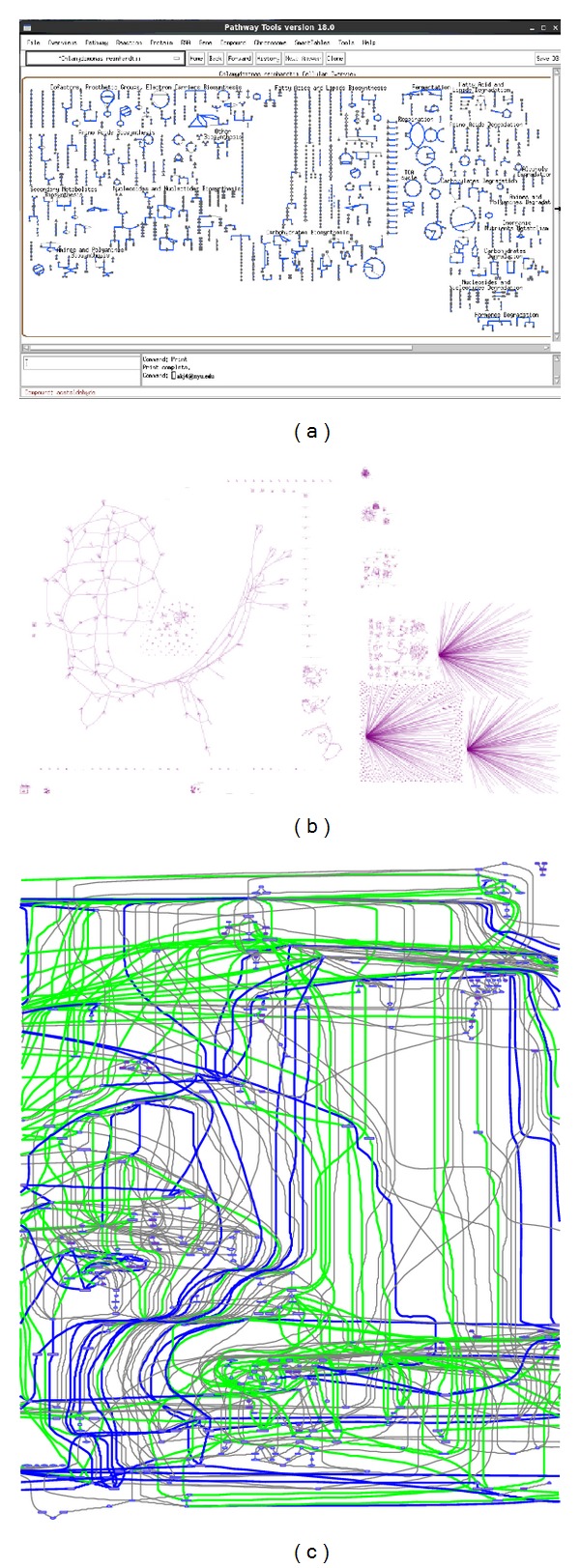
A screen shot of (a) Pathway Tools based on* C. Reinhardtii *(unpublished data) (b) Metdraw (based on the* C. reinhardtii* iRC1080 metabolic model [82]) (c) Paint4net visualization of* C. reinhardtii *central metabolism (based on iAM303 model [84]) flux distribution is shown with forward and reverse fluxes (green and blue, respectively).

Pathway Tools [6, 18] is an integrated software tool that can create in a semiautomated manner organism-specific network and pathways databases (called Pathway/Genome Database, or PGDB). The PGDBs are essentially knowledge bases that users can query and visualize. For instance, dead-end metabolite analysis and visualization of predicted reaction fluxes can be done easily under “cellular overview” option of the software (Figure 1(a)). A collection of approximately 3,530 PGDBs can be found in BioCyc, which users can visualize, manage, and analyze. Out of these 3,530 PGDBs, only 7 relate to algae (both prokaryotic and eukaryotic), namely,* Thalassiosira pseudonana*,* Nannochloropsis gaditana*,* Acaryochloris marina*,* Anabaena cylindrica*,* Anabaena variabilis*,* Synechococcus elongatus,* and* Chlamydomonas reinhardtii*. None of the aforementioned algal PGDBs are well-curated with most of them having had slight validation. One of the intensively curated PGDBs is MetaCyc [19–21], which serves as a generic knowledge base that organism-specific networks can be reconstructed from.* Homo sapiens* (HumanCyc),* E. coli* (EcoCyc), and* Arabidopsis* (AraCyc) are some examples of curated, species-specific knowledge bases that can be found in BioCyc (http://biocyc.org/). Kbase (http://kbase.us/) and Biomart [22] are other examples of knowledge bases and knowledge-management platforms that are freely available and allow integration and reconciliation of a variety of data sources.

Genome-scale metabolic reconstructions have continued to expand along with the increased availability of sequenced, annotated genomes. Recent reviews describe the timeline of the appearance of publicly available metabolic models since 1999 for eukaryotes, prokaryotes and archea, and the algorithms that were used [23, 24]. The processes require inputs from different databases and experimental validations. A standard procedure for the reconstruction of genome-scale metabolic networks has been described in detail by Thiele and Palsson [25].

The process of network reconstruction, starting from genome sequences to the finished reconstructed network, is generally time-consuming and labor-intensive. Therefore, automation of the process has been of interest. A limited number of software tools for automated reconstruction are currently available (some examples are given in Table 2); for instance, AUTOGRAPH [26], GEMSiRV [27], MicrobesFlux [28], MetRxn [29], Model SEED [17, 30], SuBliMinaL Toolbox [31], FAME [32], and RAVEN Toolbox [33] can be named. A systematic comparison between some of these platforms can be found in [34]. While draft metabolic models can be generated through such software tools, intensive manual curation is still needed to resolve errors; wrong assignments, fill gaps and reconcile inconsistencies in the generated network.

## 3. Pathway Visualization

Visualization is a powerful approach to leverage understanding of pathways and reconstructed metabolic networks. In metabolic networks, nodes represent metabolites and edges denote reactions. There are a number of web-based tools to visualize biochemical and metabolic pathways; for example, Biocarta (http://www.biocarta.com/), ExPaSy (Expert Protein Analysis System, http://www.expasy.org/), and KEGG (Kyoto Encyclopedia of Genes and Genomes) can be named; however, most are static pages with only a few resources allowing authorized users to edit the pathways. The advantages that BioCyc/MetaCyc offer compared to KEGG include the ability to carry out pathway analysis, operon prediction, or comparative pathway analysis (for more details see [35]) and visualize the results.

Cytoscape [36, 37] is a biological network visualization and data integration tool that can be used to visualize the results from FBA studies (please see Constraint Based Analysis section for information on FBA). CytoSEED [38] is a Cytoscape plug-in to visualize results from the Model SEED. Fluxviz [39] is another Cytoscape plug-in to visualize flux distribution in the molecular interaction network. VANTED [40, 41] is another data visualization and data integration tool which can be utilized as a stand-alone tool. FluxMap [42] and FBA-SimVis [43] are VANTED plug-in for visualization of metabolic flux after FBA analysis. In addition, Paint4net [44] is a tool to automatically generate maps of reaction fluxes in conjunction with COBRA toolbox (Figure 1(c)).

Most recently, MetDraw [45], a new tool for visualization of genome-scale metabolic networks, has been developed (Figure 1(b)). This tool is compatible with systems biology markup language (SBML) file inputs and allows export of the map image as SVG files. It also allows visualization of metabolomics and reaction fluxes added to gene-protein expression data and overlays all of them on the reconstructed network map. The range of file formats available for data export render the postmodification of the maps, with commonly used image editing software, a simple task.

## 4. Model Refinement and Gap Filling

Although the generation of metabolic network models has been gaining momentum, these models may not provide a complete or accurate representation of metabolism. Particularly, automated modeling has allowed the faster generation of network models, yet reconciliation between the model itself and the biochemical and genomic data is invariably needed. Such model refinements lead to a more accurate reconstruction, allowing more accurate downstream analyses. A common step in such reconstruction refinements is filling reaction gaps to decrease the numbers of dead-end metabolites and enhance the network connectivity. Several tools and algorithms have been set in place to address gap finding and gap filling in metabolic network reconstructions. Some of these tools include, but are not limited to, Gapfill, MEP, GrowMatch, BNICE, and the hole filler in Pathway tools.

### 4.1. Gapfind and Gapfill

These tools have been developed using two distinct algorithms that initially identify (Gapfind) what the authors have called a “no production” or “no consumption” metabolites [46] through analyzing the production or consumption fluxes in the metabolic model. Subsequently, the identified no production/consumption metabolites are considered as “gaps” and the Gapfill algorithm will attempt to fill them through four major ways. Initially, the algorithm will consider all of the available reactions in the model and reverse them; it will then attempt to import reactions that involve the metabolites from well-curated databases such as MetaCyc [10]. Lastly, it will attempt to fill these gaps by adding transport reactions either internal transport ones, as in from one cellular compartment to the other, or external transport reactions that can either take from or excrete to the extracellular medium.

### 4.2. MEP and Pathway Tools Hole Filler

On the other hand MEP and Pathway Tools hole filler represent an alternative approach that tackles the gap filling issue identifying missing genes rather than missing reactions, and these tools achieve this goal using expression data and species homology, respectively. As such, this will eventually lead to the expansion of the reconstructed model to include more genes and enzymes and possibly rewire the connectivity of the network [47, 48].

### 4.3. GrowMatch

This tool has been developed as a model refinement tool rather than a gap filler tool where the aim of such an application would be to reconcile inconsistencies between metabolic model predictions* in silico* and growth data* in vivo*. This computational tool can suggest suppression of specific genes to resolve what is referred to as Growth No Growth (GNG) inconsistencies and alternatively adds functionalities to genes to resolve No Growth/Growth (NGG) inconsistencies [49].

### 4.4. BNICE

It is a framework that considers specific pathways rather than the full-scale model and allows for the optimization of the pathways. It identifies all possible chemical compounds that can be produced by the reactions and enzymes of the pathway [50]. Although this tool is not a model refinement tool* per se*, the outcome of the pathway optimization can ultimately lead to provisional addition of compounds to the metabolic model and subsequent searches (independently from the tool) for the corresponding genes to provide genomic evidence for the pathway. This approach is similar in outcome to the Gapfind/Gapfill approach.

All of the above and many more tools are of critical importance in the manual curation of metabolic network models. Although the above-mentioned tools ultimately lead to a similar outcome, each may present unique advantages and has specific requirements (Table 3). The choice and use of such tools would thus lead to a higher quality reconstruction and most importantly a higher predictive power.

## 5. Constraint-Based Modeling, FBA, and Integration of Expression Data

Subsequent to generation of well-curated metabolic network models of organisms, several downstream applications can be used to explore the emergent system's properties. Having a network set in place, the fluxes of each of the component reactions can be evaluated and moreover modified in an attempt to increase or decrease the production or consumption of key metabolites. In the case of algal biofuel optimization, it is of high interest to achieve directional overproduction of lipids that constitute the basis for algal biofuels. Making use of the known metabolic networks and via a constraint based modeling approach, the identification of genes, pathways, and knockout strategies, that interfere or alter, the expression profiles relevant to production of enzymes related to lipid synthesis and metabolites involved in lipid synthesis pathways is readily achievable. This can be done through a number of computational tools with the outcomes evaluated* in silico *using flux balance analysis (FBA) [51] and further validated by* in vivo* experiments.

FBA constrains the metabolite fluxes and their biochemical reactions by four main parameters: mass conservation, thermodynamics (reaction reversibility), steady state assumption for internal metabolite concentrations, and nutrient availability. Based on these constraints, reaction boundaries are set, and a system of linear differential equations is solved with a biologically relevant objective function optimized. The solution space for an FBA can be reduced in size by more constraints and boundaries imposed on reactions and fluxes where the optimal flux distribution achieving the optimized function is a feasible solution for the problem.

Some of the available tools and algorithms that are able to perform such tasks include (but are not restricted to) Optknock, Optstrain, Optflux, MTA, iMAT, BioMet toolbox, PROM, GIMME, E-Flux, MADE, SIMUP, and TIGER, with some allowing the integration of expression data to the metabolic model. These tools are described below.

### 5.1. GIMME, iMAT, and MADE

Gene inactivity moderated by metabolism and expression (GIMME) [52] is a tool that allows for the integration of expression data to metabolic networks yet optimizing the functionality of the model towards a set objective function by minimizing the use of reaction categorized as inactive. GIMME reduces the sets of reactions to a binary on/off mode whereas each reaction flux is compared to a set threshold and deemed “off” if the flux does not reach that value [53, 54]. Similarly, integrative metabolic analysis tool (iMAT) [55] performs the same task as GIMME in such a way that transcript levels of genes are compared and the corresponding reactions are then assigned value of −1, 0, and 1 to refer to low, moderate, or high levels of expression. Further ahead, the algorithm will then optimize the model to make use of as many reactions having “1” coefficient and decreases the reactions with “−1” coefficient in order to achieve a set objective function. Here too, a threshold needs to be set for expression data comparison to be done. As both iMAT and GIMME require a manually set threshold, this gives rise to biases. In an attempt to evade such a complication, MADE [56], or metabolic adjustment by differential expression, has been developed to carry out similar tasks as the previous two tools yet without the need of manual assignment of a threshold. It will rather require as input expression data originating from more than one condition and will then comparatively, based on the differential expression of each of the genes under each of the conditions, set a threshold based on which the reactions will then be reduced to binary on/off code [53, 54].

### 5.2. E-Flux

While the above-mentioned tools allow the incorporation of expression data to metabolic model reconstructions and subsequently allow optimization of these models towards a set objective function by suppressing reactions categorized as inactive or of low activity, E-flux allows for this optimization through constraining the upper bounds of the metabolite fluxes based on the expression data by imposing tight constraints on metabolites and reactions where the fluxes will not reach a set value and vice versa [57].

### 5.3. Optknock, Optstrain, and Optflux

These tools have been used to identify gene knockout strategies (Optknock) [58] that lead to the overproduction of a target metabolite or overexpression strategies (Optstrain) [59] that result into an optimized strain with respect to a set objective function. Optflux on the other hand uses evolutionary algorithms and the previously mentioned Optknock algorithm to identify metabolic engineering targets as well as a range of other applications from phenotype simulations to metabolic flux analysis and calculation of elementary flux mode [60].

### 5.4. BioMet Toolbox

It is a web-based resource that can be used to perform stoichiometric analyses and integration of transcriptome and interactome data to a metabolic network. It also allows performing linear programming simulations, optimizing for an increased or decreased growth rate, as well as substrate consumption and production. Single or double knockout simulations can also be achieved as well as the detection of key metabolites around which high transcriptional activity is noted [61].

### 5.5. MTA

Metabolic transformation algorithm [62] is an alternative approach that leads to the prediction of gene knockout strategies able to shift the metabolism of a cell and alter its state from a “source” state to a “target” state. Gene expression profiles are used in order to predict knockouts that modify the flux distribution of the source state in a way to match the desired target state.

### 5.6. TIGER

It is a toolbox that can be used to integrate expression, metabolic and regulatory information into a genome scale model. It also accounts for gene-protein-reaction associations and couples it with its regulatory profile. One of its added values is its ability to identify model inconsistencies and thus it allows for a modification of the reconstructed network above and beyond being an integration tool [63].

### 5.7. SIMUP

Most recently, this algorithm was reported offering one unique feature with respect to all of the above introduced tools. The algorithm aids in identifying metabolic engineering strategies that can force the cell to coutilize two different sugar substrates thus, in effect, placing the cell in a “synthetic survival” state in a way that the cell is now forced to metabolize two different sugars simultaneously instead of preferentially consuming one. The net effect can be to simplify the fermentation cycle [64, 65].

In the context of biofuels, all of the above algorithms and tools present huge potential for achieving higher production of the desired bioproducts in microorganisms. The preferential use of one tool over the other may depend on the nature of data available rather than the ultimate goal (Table 4). The identification of knockout strategies that could alter the lipid metabolism by overproducing it, or the detection of highly regulated key metabolites in the lipid pathway, or even achieving a strain able to coutilize two separate sources of energy for its survival, all represent promising outcomes of such applications and several attempts have been already made making use of such algorithms (the results could be found in more detail in the published articles [66, 67]).

## 6. Omics Data Integration Tools

Beyond the integration of expression data to network models, a deeper understanding of the functional model requires further integration of proteomics, metabolomics, fluxomics, and phenotypic data with transcriptomics data. Computational tools and algorithms have been recently set forth to achieve the aforementioned integrations. IOMA, MASS, and MBA are examples of such endeavors.

### 6.1. IOMA

Integrative omics-metabolic analysis is an algorithm that allows the integration of metabolomics and proteomic data to the metabolic network model and also evaluates the kinetics of the reactions included [68].

### 6.2. MASS

Mass action stoichiometric simulation [69] achieves integration of fluxomic data on top of the metabolomics and proteomics data sets which leads to the dynamic reconstruction of the model in place.

### 6.3. MBA

Model-building algorithm [70] has been recently reported with an added feature allowing it to also integrate phenotypic data on top of all the above-mentioned omics data sets, thus potentially leading to tissue-specific model reconstruction.

With respect to phenotypic data, one interesting tool that may generate such type of data and can be used in conjunction with MBA, for example, is the Biolog phenotype microarray technology [71, 72]. The Biolog is a powerful technology providing high-throughput quantitation of phenotypic data, useful in identifying additional biochemical assays and improving a metabolic model reconstruction.

The phenotype microarray (PM) technology developed by Biolog (Hayward, CA, USA) can be used for the phenotypic analysis. Biolog is an* in vitro* assay that measures the respiration of cells as a function of time in hundreds of microwells simultaneously. Each PM plate contains 96 wells seeded with different metabolite and monitored automatically over time via the OmniLog machine. Metabolite utilization within the cell is determined by the amount of color development produced by a tetrazolium-based redox dye. Various 96-well metabolite plates (or PMs) can be used to measures carbon source, nitrogen, sulfur, and phosphorus utilization phenotypes. Some plates were used to test for osmotic/ion and pH effects. Data analysis is performed using the opm software package [73]. The Biolog technology has also been successfully used to fill gaps in metabolic networks to enhance models [74].

## 7. Bioengineering, Parts and Circuits

With all of the above tools readily available to use and many others currently in use but not described in this review, the identification of new pathways and reactions has been made easier than ever before. In the context of bioengineering, the significance of these computational tools is in guiding wet-bench experimental design as opposed to providing solely theoretical insight into the system as a whole. More specifically, with regard to biofuel production, the identification of knockout strategies or differential expression of genes or enzymes that might lead to overproduction of biofuels would be only of theoretical value if not coupled with more applicable approaches to achieve the targets* in vivo*. This is where the contributions of synthetic biology approaches are of crucial importance and significance. Once the target pathways have been identified, the parts forming those pathways, in engineering terms, are to be made available in order to mimic the cell metabolic circuitry and alter it. Parts are defined as genes and ribosomal binding sites, promoters, terminators, and polymerases [75]. Most recently, Talebi et al. have successfully achieved a 12% increase in the total lipid content of the microalgae* Dunaliella salina,* transforming it witha bioengineered plasmid comprising specific parts, genes, and inducible promoters, driving the cellular carbon flux into the fatty acids biosynthesis pathway [76].

**Figure 2:**
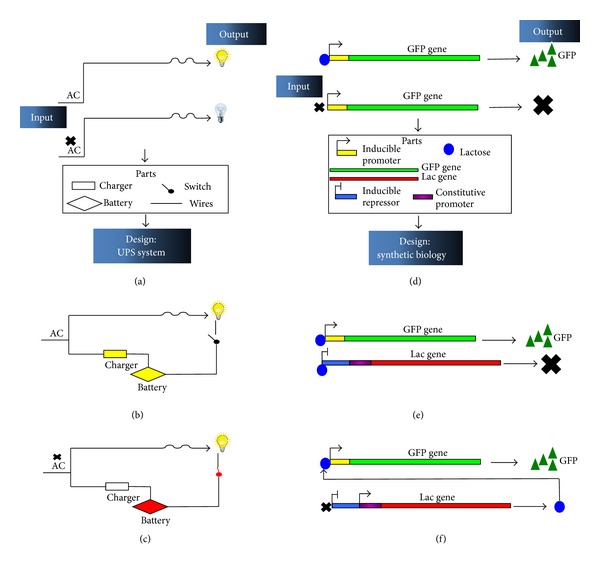
A schematic representation of a comparative design of electrical and analogous biological circuit. (a) and (d) represent the initial states of the circuits in presence and absence of the input. (b), (c), (e), and (f) represent the designed circuit, addressing the issue raised by the “wild type” design of (a) and (d) when the input signal is interrupted or is not present.

Biological circuits are furthermore defined as a designed device made out of a set of parts and engineered in a way to confer an added functionality to a system.  Figure 2 illustrates, in a comparative approach to electrical circuits, what a newly designed biological circuit can achieve. A number of biological circuits have been previously realized [77–79] and genetic parts are now made available through a number of databases such as the MIT Registry of Standard Biological Parts' (http://partsregistry.org/). A more in-depth review on the tools and applications that lead to the design of circuits was published by Marchicio et al. and can be referred to for more details [80].

## 8. Emerging Algal-Specific Computational and Experimental Resources

**Figure 3:**
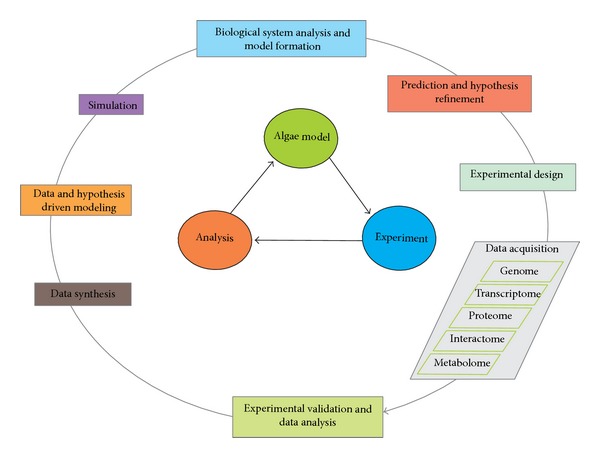
A conceptual representation of algal model reconstruction and refinement, integrating various sets of omics data and experimental validation of predictions (based on Manichaikul et al., 2009 [84]).

Optimizing algae for biofuel production requires a deep understanding of algal metabolic networks with genomic, fluxomics, proteomics, and metabolomics data integration.  Figure 3 conceptualizes an integrative approach to build, refine, and validate an algae based metabolic model with predictive power to guide potential bioengineering targets aimed at optimizing algae for biofuel production.

Furthermore, a better understanding of the biological system through functional modeling using data generated from the sequencing technologies is still one of the research challenges. Functional modeling requires gene ontology (GO) annotation for enrichment analysis. GO enrichment analysis tools identify GO terms with statistical significance in the reference set. Algal Functional Annotation Tool is the algae-specific genome annotation tool that uses gene lists from AUGUSTUS, JGI, or phytozome gene models for* Chlamydomonas reinhardtii* and* Chlorella *NC64A [81] to perform functional term enrichment. This functional annotation tool provides analytical power for interpretation of obtained large-scale experimental data.

**Figure 4:**
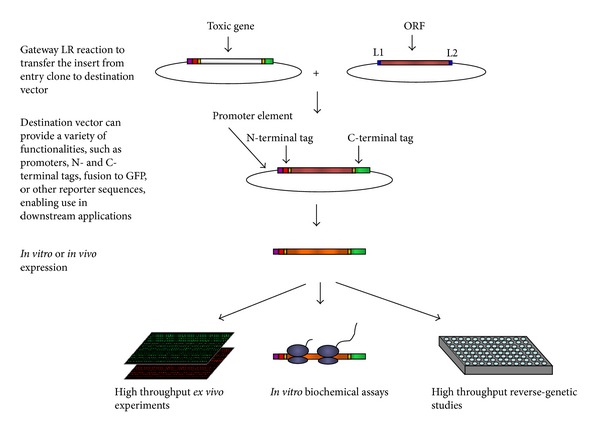
A summary figure representing recombinational transferring of an ORF from a gateway vector in which the initial cloning was done into destination vectors for downstream applications, including high throughput experiments and biochemical assays. Once an ORF is cloned into an “entry vector,” the ORF can easily be transferred into many “destination vectors” with desired expression capabilities and tags [87].

Interestingly, a new approach in bioengineering, transcription factor engineering approach (TFE) [67], is regarded as a highly promising approach and considers transcription factors as parts able to modify biological circuits. An ongoing work (in the authors' laboratory) is now attempting to systematically clone transcription and chromatin factors (TF and CF) of* C. reinhardtii* thus making available to the scientific community a full library of TF and CF parts that can easily be introduced as part of a new design.  Figure 4 represents one step further downstream the initial cloning and describes the transfer of cloned ORFs from the entry vector to the destination vector of choice. These ORFs can be considered as potential parts to be used in bioengineering endeavors when model-based predictions call for their use. Furthermore, the metabolic ORFeome of* C. reinhardtii *has been previously generated and the reconstruction of its central metabolic network has been done [82–84]. Following that, genome-scale reconstructed networks of* C. reinhardtii* were released accounting for around 2000 reactions and their associated genes and metabolites [82, 85]. Added to these models, a PGDB for* C. reinhardtii *has been made available as ChlamyCyc [86] making use of Pathway Tools platform and thus making the investigations of the metabolic and regulatory networks of such algae far more at hand. Prior and in parallel to these advances a species specific resource, Chlamydomonas Resource Center (http://chlamycollection.org/), has served the algal community offering a library of Chlamydomonas strains amongst other parts and tools, which provide needed resources for experimental protocols targeting various aspects of algal biology, including the metabolism of lipids and biofuels in this organism.

## 9. Conclusion

The above reviewed computational tools and approaches in conjunction with the high interests of the scientific community in synthetic biology offer a new perspective in accelerating biofuel production and microalgal optimization research. The pressing economical and environmental challenges of the use of fossil fuels will furthermore lead to a positive selective pressure towards the use of these strategies aiming at the optimization of biofuel producing strains. A large set of biofuel types can serve as alternative energy sources which currently include ethanol, n-butanol, iso-butanol, short chain alcohols, short chain alkanes, biodiesel (FAMEs), and fatty alcohols. These tools and applications are promising yet much more optimizations need to be achieved in order for biofuel production to compete with available fossil fuels. With the “green revolution” and the more environmentally conscious population, we expect this field to expand significantly in the coming years, building on the available resources for systems and synthetic biology and achieving the generation of strains optimized for biofuel production.

## Figures and Tables

**Table 1:** Databases and tools for metabolic network reconstruction.

Database	Link
Algal Functional Annotation Tool	http://pathways.mcdb.ucla.edu/algal/index.html
BiGG	http://bigg.ucsd.edu/
BioCyc	http://biocyc.org/
Biomart	http://www.biomart.org/index.html
BRENDA	http://www.brenda-enzymes.info/
COBRA	http://opencobra.sourceforge.net/openCOBRA/
ExPASy	http://www.expasy.org/
KBASE	http://kbase.us
KEGG	http://www.genome.jp/kegg/
Model SEED	http://www.theseed.org/wiki/Main_Page
MetaCyc	http://metacyc.org/
Pathway Tools	http://pathwaytools.org/
Reactome	http://www.reactome.org/PathwayBrowser/
UniProt	http://www.uniprot.org

**Table 2:** Selected software for genome-scale metabolic reconstruction (adapted from Liao et al., 2012 [27]; Agren et al., 2013 [33]; and Hamilton and Reed, 2014 [34]).

	RAVEN	Model SEED	SuBliMinal	GEMSiRV	Pathway Tools	COBRA toolbox
Input	Annotated genome sequence	Genome annotated in RAST	Species name	Model in sbml format	Annotated genome sequence	Model in sbml format

Reference databases	KEGG	SEED	KEGG, MetaCyc	KEGG	MetaCyc	N/A

Interface	MatLab	Web	Command line	Software	Web, software	MatLab

License	Free (requires a MatLab license)	Free	Free	Free	Free for academic and government use	Free (requires a MatLab license)

Simulation	Yes	Yes	No	Yes	Yes	Yes

Visualization	Yes	Yes (with Cytoscape plug-in)	No	Yes	Yes	Yes (with plug-in)

**Table 3:** A comparative table contrasting some of the major model refinement tools.

	Gapfind and Gapfill	GrowMatch	BNICE	MEP	Pathway Tools hole filler
Require a reconstructed metabolic model	Yes	Yes	No	Yes	Yes

Additional requirements	External databases, e.g., MetaCyc	Requires *in vivo* data collection	Requires the translation of reactions and substrates into mathematical matrices	Requires expression data analysis	Requires species homology analysis

Refinement strategy	Identifies missing reactions or reverses available reactions	Suppresses genes or adds functionalities associated with genes in the initial model to reconcile the model with *in vivo* data	Optimizes pathways in a way that can provide feedback into the model adding compounds and substrates	Identifies missing genes in the model	Identifies missing genes in the model

**Table 4:** A comparative table contrasting major constraint based modeling tools (adapted from Blazier and Papin 2012 [53]).

	GIMME	iMAT	MADE	E-Flux	SIMUP	MTA
Description	Determines sets of active versus inactive reactions comparing expression levels to a set threshold optimizing the model towards a set objective function	Categorizes reactions into high, moderate, and low expression and solves mathematical equation to optimize for an objective function	Establishes a differential expression profile using several datasets originating from different growth conditions	Sets upper bounds for lowly expressed reactions using an externally set threshold to evaluate expression data sets	Identifies bioengineering strategies that force the cell to coutilize substrates achieving a state of “synthetic survival”	Predicts gene knockout strategies that would alter the metabolic fluxes in a cell in order to achieve the objective function assumed

Advantages	Requires one set of expression data	Requires no knowledge of metabolic functions	Requires no externally set threshold for expression levels	Requires no reduction of expression data to an on/off categorization	Achieves the coutilization of two sugars	Categorizes cell metabolism as “source” or “target” with no necessary *a priori* knowledge of functionalities

Disadvantages	Requires an externally set threshold for mRNA transcript values	Categorizes genes into high, moderate, and low expression	Requires more than one dataset of expression data to establish differential expression profiles	Sets an upper bound on fluxes using a specific function converting expression data	So far only applicable to sugars	Requires gene expression profiles in order to identify knockout strategies
